# The perceived duration of expected events depends on how the expectation is formed

**DOI:** 10.3758/s13414-022-02519-x

**Published:** 2022-06-14

**Authors:** Blake W. Saurels, Derek H. Arnold, Natasha L. Anderson, Ottmar V. Lipp, Kielan Yarrow

**Affiliations:** 1 School of Psychology, The University of Queensland, Brisbane, QLD Australia; 2 School of Psychology, Queensland University of Technology, Brisbane, QLD Australia; 3 Department of Psychology, City, University of London, London, UK

**Keywords:** Prediction, Expectation, Oddball

## Abstract

Repeated events can seem shortened. It has been suggested that this results from an inverse relationship between predictability and perceived duration, with more predictable events seeming shorter. Some evidence disputes this generalisation, as there are cases where this relationship has been nullified, or even reversed. This study sought to combine different factors that encourage expectation into a single paradigm, to directly compare their effects. We find that when people are asked to *declare* a prediction (i.e., to predict which colour sequence will ensue), guess-confirming events can seem relatively protracted. This augmented a positive time-order error, with the first of two sequential presentations already seeming protracted. We did not observe a contraction of perceived duration for more probable or for repeated events. Overall, our results are inconsistent with a simple mapping between predictability and perceived duration. Whether the perceived duration of an expected event will seem relatively contracted or expanded seems to be contingent on the causal origin of expectation.

## Introduction

The perceived duration of identical repeated events within a sequence can seem shorter than a surprising ‘oddball’ that breaks the train of repetition (the temporal oddball effect; Pariyadath & Eagleman, 2007; Saurels et al., 2019; Schindel et al., 2011; Tse et al., 2004). This effect can be separated from the effects of low-level sensory adaptation (Schindel et al., 2011), and it benefits from consistency in terms of the spatial location of presentations (Birngruber et al., 2015; Cai et al., 2015; Johnston et al., 2006). It remains unclear precisely what causes this effect and how it relates to other distortions of perceived time, such as time-order errors (whereby one event in a pair can seem longer; Eisler et al., 2008; Hellström, 1985).

There is some evidence that the temporal oddball effect might occur not only for physically identical repeated events, but also for other predictable inputs. For example, statistically probable events can seem shorter (Tse et al., 2004; Ulrich et al., 2006). From this, one might reasonably hypothesise a similar relationship between perceived duration and other predictive processes – potentially, visual events that confirm any form of short-term prediction might seem shorter.

Other research has, however, raised doubt about the generality of the impact of predictability upon perceived duration. For instance, Cai et al. (2015) had participants view sequences of shapes or numbers that were predictable due to repetition, or because of a ‘high-level’ expectation effect (e.g., they were statistically more probable, or they were part of an ‘over-learned’ sequence, such as 1-2-3-4-5). They found that only a repetition of physically identical events reduced perceived duration, inconsistent with the generalisation that predictable events should always seem shorter.

Research from Birngruber et al. (2015) suggests a large part of the classic temporal oddball effect is driven by an absence of knowledge about when, or for which event(s), a temporal judgement will be required. When this is controlled for, by flagging which event within a train will need to be judged, the perceived duration difference between repeated and oddball events was reduced (Birngruber et al., 2015, Exp. 3) or eliminated (Exps. 1 and 2, although spatial location varied for these, and this appears necessary for the effect, see Cai et al., 2015, and Johnston et al., 2006). This does not rule against the existence of a repetition-based contraction of perceived duration, as these have consistently been observed in other experiments – even when the to-be-judged event has been predictable (e.g., Cai et al., 2015). It does suggest that many of the temporal oddball effects that have been reported may have been produced in part by awareness of when a perceptual judgement would be required (e.g., Pariyadath & Eagleman, 2007; Saurels et al., 2019; Schindel et al., 2011; Tse et al., 2004), instead of solely by an effect of ‘repetition’. Of course, this effect could also result from modulating awareness/attention, but the trigger would be the event itself, rather than a pre-emptive motivation to up- or down-regulate the processing of certain events.

Further complicating the relationship between predictability and perceived duration, Birngruber et al. (2018) have observed that events that confirm explicit, declared predictions can appear *longer*. These researchers had participants vocalise a guess about the colour of an upcoming image. When the guess was correct, the coloured image seemed relatively protracted (participants classified events as either ‘rather short’ or ‘rather long’). This is a reversal of the expectation/perceived duration relationship that manifests in many temporal oddball experiments – where expected events seem to have a shortened duration (Pariyadath & Eagleman, 2007; Tse et al., 2004; Ulrich et al., 2006; although see Cai et al., 2015). Events that confirm explicit, non-declared predictions can also seem to have a relatively longer duration. This can be encouraged by making someone more certain that a duration judgement will be required (Grondin & Rammsayer, 2003; Wehrman et al., 2020), or by cueing the location (Enns et al., 1999) or modality (Mattes & Ulrich, 1998) in which a to-be-judged event will occur.

Matthews (2015, 2016) has suggested that the temporal oddball effect might be shaped by an interplay between bottom-up and top-down influences – repetition might weaken bottom-up signalling, thereby reducing perceived duration (see Pariyadath & Eagleman, 2007), whereas high-level expectations could trigger an enhanced response to anticipated inputs, thereby encouraging an expansion of perceived duration. The impact of guessing (Birngruber et al., 2018) could be incorporated within this framework, in a manner reminiscent of confirmation bias – the tendency to seek out, and preferentially attend to, information that is consistent with our expectations (Bundesen, 1990; Nickerson, 1998; Rajsic et al., 2015). Declared prediction-confirming events might trigger an enhanced analysis of input, and thereby encourage a protracted perceived duration.

According to this conjecture, the relationship between expectation and perceived duration should be multifaceted, with different outcomes driven by different *types* of prediction (Downing, 2007). On the one hand, events that are expected due to repetition, and/or statistical likelihood, should seem to have a relatively *shortened* perceived duration (Pariyadath & Eagleman, 2007; Saurels et al., 2019; Schindel et al., 2011; Tse et al., 2004; Ulrich et al., 2006), whereas events that are expected because they corroborate a declared prediction should have a relatively *protracted* perceived duration (Birngruber et al., 2018).

While several factors that promote predictability have been identified, their impacts can be opposite, and they have not been combined in a study (i.e., we are not aware of any prior study that has combined repetition and guessing). Previous work has also been inconsistent as to whether statistical regularities in the environment, beyond basic repetition, modulate perceived duration (Cai et al., 2015; Tse et al., 2004; Ulrich et al., 2006). Moreover, factors that promote an expectation have often been confounded in previous studies (e.g., repetition-violating events are often statistically improbable). In this study we aimed to pit three factors that promote predictability against each other within a single paradigm, while controlling for the extraneous influence of uncertainty. Specifically, we examine the effects of repetition, statistical probability, and guessing – all within a presentation protocol consisting of sequential event couplets. We hypothesise that repetition and an increased likelihood of presentation should encourage a *contraction* of perceived duration, whereas events that confirm a declared prediction should seem to have a relatively *protracted* duration.

## Methods

Experimental procedures, participant numbers, exclusion criteria and analyses for this experiment were pre-registered (https://aspredicted.org/8nb3c.pdf).

### Participants

Forty-seven participants were recruited for testing via a research participation scheme at the University of Queensland (11 participants received course credit for participation, and the remaining 36 participants received $20 AUD compensation). All reported having normal or corrected-to-normal visual acuity. Of the tested participants, five were excluded from analysis based on pre-registered criteria (see *Results* section), so the number of participants subjected to formal statistical appraisal (42; 15 males) reached our pre-registered target *N*. Ages ranged from 18 to 30 years (*M* = 22, *SD* = 3). This experiment was approved by the University of Queensland ethics committee and was conducted in accordance with the principles of the Declaration of Helsinki.

### Stimuli and apparatus

Stimuli were coloured circles (red or green), with a diameter subtending ~23° of visual angle. Green and red circles were matched in luminance (green circles CIE: 0.2858, 0.5939, 8.9311; red circles CIE: 0.6337, 0.3117, 8.9311). Stimuli were presented on a calibrated 20-in. CRT SyncMaster 1100p-Plus monitor, driven by a Cambridge Research Systems ViSaGe stimulus generator and custom Matlab R2007b (The MathWorks, Inc., 2007) software. The monitor had a resolution of 1,024 × 768 pixels and a refresh rate of 100 Hz. Participants viewed stimuli from 57 cm, from directly in front of the monitor with their chin placed on a chin rest.

### Procedure

**Fig. 1:**
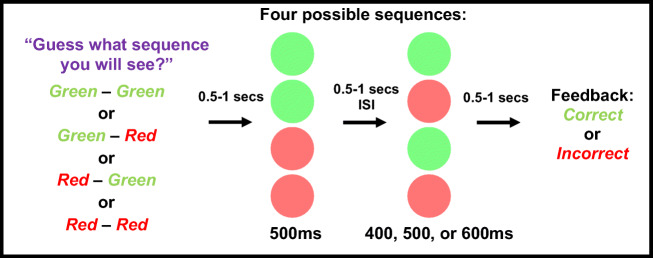
Graphic depicting the basic trial sequence. The likelihood that participants got their first guess colour correct was set to 80%. There were three experimental blocks, each with a different ratio of repeat to change sequences (80:20, 20:80, and 50:50; counter-balanced across participants)

Each trial consisted of two sequential circle presentations (Fig. 1). The first persisted for 500 ms, whereas the second was 400, 500, or 600 ms (equiprobable and randomised), to create a sense that test durations were physically varying. Presentations were separated by a 500- to 1,000-ms inter-stimulus interval (ISI), with the precise ISI duration determined at random on a trial-by-trial basis.

To manipulate repetition, circles on each trial were either the same colour (Green then Green, or Red then Red – the Repeat condition), or the second colour was different (Green then Red, or Red then Green – the Change condition).

To manipulate statistical likelihood, the experiment was split into three blocks of 80 individual trials, with each block containing a different ratio of Repeat to Change trials: 80:20, 20:80, or 50:50. Trial blocks were presented in an order that was counter-balanced across participants. More probable trials of unbalanced blocks constituted the ‘Probable’ condition, and less probable trials the ‘Improbable’ condition. Trials within balanced 50:50 blocks constituted the ‘Balance Probability’ condition. Participants were informed of the probability that would prevail in each block before the first trial.

Declared predictions were measured by having participants *predict* what sequence of colours they were about to see (e.g., red – red, or red – green). To encourage participants to engage in this task, they were told there would be an underlying pattern to the order of sequence presentations that they might discern if they paid close attention. To reduce discouragement due to poor guessing, the first flash colour was set to the participants’ first ‘guess’ with a probability of 80% – so, participants were seldom wrong in their guess regarding the first colour.

At the end of each trial, participants reported if they thought the second flash had been longer (right click) or shorter (left click) than the first using a mouse. Each participant completed three practice trials at the beginning of the experiment, to familiarise themselves with the task and trial sequence. The duration of the second flash on these trials was 200, 500, or 800 ms, making it easier on average for participants to judge which flash had seemed longer, and helping the experimenter to verify that the participant had understood task instructions.

## Results

Data were collected and organised using Matlab R2021b (The MathWorks, Inc., 2021) and analysed using a Bayesian analysis toolbox built by (Krekelberg, 2021), with a Cauchy prior width of 0.707.

Three participants were excluded from formal analyses as they failed to report that the second flash had seemed longer more often when it was physically longer (600 ms), relative to when it was shorter (400 ms) than the first (500 ms) event. Two more participants were excluded from formal data analyses because they guessed the same colour sequence on more than 15 consecutive trials – suggesting they might not have engaged with the prediction-declaring task. All participants showed a bias to make intuitive guesses that reflected the statistical likelihood of presentations in unbalanced blocks of trials (i.e., they predicted more Repeat trials when these were more probable, and more Change trials when these were more probable).

**Fig. 2:**
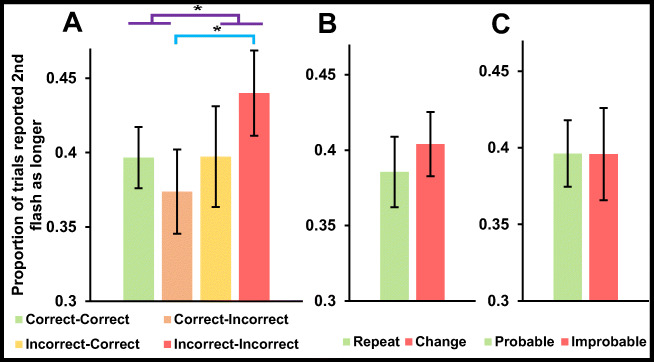
All three graphs display the proportion of trials on which participants reported the second flash had appeared for longer than the first. (**A**) Trials on which participants guessed both colours correctly (green), just the second colour correctly (yellow), just the first colour correctly (orange), or neither correctly (red). Note that participants were more likely to report that the second flash had seemed shorter after they had guessed the colour of the first flash correctly. (**B**) Trials on which participants saw two flashes of the same colour (Repeat trials: green) or two differently coloured flashes (Change trials: red). (**C**) Trials on which participants saw a statistically probable sequence (green) or a statistically improbable (red) sequence. In all cases, error bars depict ±1 standard error

We first tested for a time-order error. Participants were more likely to report that the second flash had seemed shorter (*M* 61%, *SD* 14%) than longer (*M* 39%, *SD* 14%; *t*_41_ = 4.904, *p* < .001, *d* = 0.757). A Bayes factor analysis of these data indicated strong evidence for the alternative hypothesis – that there would be a debut effect (*BF*_10_ > 1000; see Fig. 2 – and note that across all conditions < 50% of second stimulus presentations seemed longer than the first).

We initially planned to compute the proportion of trials each participant reported the second flash as longer individually for each sub-condition (e.g., independently calculate proportions for correctly guessed repeats from blocks where these were probable, and correctly guessed repeats from blocks where these were improbable), and then subject these data to a three-way ANOVA. This method provides an equal weighting in analyses to each sub-factor, nullifying the confounding of guess outcome and sequence probability – as participants were more likely to guess the more probable sequence option (i.e., under this method, probable and improbable event sequences contribute equally to estimates of the effect of guessing, and correct and incorrect guesses contribute equally to estimates of the effect of event type). The cost, however, is that this overweights sub-conditions with very few trials. This analysis plan proved to be unsuitable in practice, as some sub-conditions (e.g., correctly guessed, statistically improbable sequences) often contained no trials, or just 1–2.

We instead analysed each predictive effect individually. We first conducted a 2 (first flash correct vs. incorrect) × 2 (second flash correct vs. incorrect) repeated-measures ANOVA to determine the effect of guess outcome on apparent duration. We found no main effect for second flash outcome (*F*_1,41_ = 0.42, *p* = .521, with a Bayes factor of *BF*_10_ = 0.199 indicating substantial evidence for the null hypothesis – that there would be no conditional difference; see Fig. 2A). We did, however, find that when participants got the first flash correct, they were more likely to report the second flash as shorter (*F*_1,41_ = 4.43, *p* = .042, with a Bayes factor of *BF*_10_ = 1.327 indicating anecdotal evidence for the alternative hypothesis – that there would be a conditional difference). This main effect was qualified by an interaction (*F*_1,41_ = 4.98, *p* = .031, with a Bayes factor of *BF*_10_ = 1.75 indicating anecdotal evidence for the alternative hypothesis – that there would be an interaction), such that correctly guessing the first flash colour only made the second flash seem shorter when the second flash colour was incorrectly guessed (*F*_1,41_ = 14.07, *p* < .001, with a Bayes factor of *BF*_10_ = 51.780 indicating very strong evidence for the alternative hypothesis – that there would be a conditional difference).

We then conducted two frequentist and Bayesian paired *t*-tests to determine the impact of sequence pattern (Repeat vs. Change) and sequence probability (Probable vs. Improbable) on apparent duration. These comparisons revealed no evidence for conditional differences (*sequence outcome*: *t*_41_ = 1.445, *p* = .156, *d* = 0.223, with a Bayes factor of *BF*_10_ = 0.437 indicating weak evidence for the null hypothesis – that there would be no difference contingent on trial type – see Fig. 2B; *statistical likelihood*: *t*_41_ = 0.022, *p* = .983, *d* = 0.003, with a Bayes factor of *BF*_10_ = 0.167 indicating moderate evidence for the null hypothesis – that there would be a difference contingent on statistical likelihood – see Fig. 2C).

### Guessing behaviour

Participants guessed repeats ~86.2% (*SD* = 8%) of the time in blocks where repeats were more likely, and therefore slightly overestimated their likelihood (*BF*_10_ > 1000, comparing guessing likelihoods to the true likelihood of 80%). Participants guessed changes ~83.3% (*SD* 10.2%) of the time in blocks where changes were more likely, and there is only anecdotal evidence that this rate was different from the true likelihood (*BF*_10_ = 1.173). In blocks where repeat and change pairings were equally likely, participants guessed repeat options ~49.4% (*SD* 7%) of the time. A Bayes factor analysis revealed moderate evidence that participants were performing at chance on these blocks (*BF*_10_ = 0.195, comparing guessing likelihoods to the true likelihood of 50%).

Percent correct in unequal probability blocks (*M* 56.1%, *SD* 5%) was greater than in the equal probability block (*M* 39%, *SD* 5.3%, *t*_41_ = 14.32 *p* < .001), with a Bayes factor analysis revealing decisive evidence for the alternative hypothesis, that there would be a difference (*BF*_10_ > 1000).

One might reasonably ask if participants attempted to predict the second event colour in unbalanced blocks of trials, or if they had simply always chosen the more likely sequence contingent on their first guess (i.e., in blocks of trials where repeats were more likely, a participant might guess at the first colour, and then always predict that colour would repeat). This ‘no attempt to guess at the second colour’ strategy would deliver a percentage correct on unbalanced blocks of 64%. To explain, by design participants correctly guessed the first colour on 80% of trials and would then incorrectly predict the more likely event would ensue on 20% of these trials (i.e., 0.8 – (0.8 × 0.2) = 0.64). If, however, participants had attempted to guess at the second colour – informed by instructions regarding the more likely sequence (on 80% of trials), we would predict that they should obtain an overall percentage correct on unbalanced blocks of 54%. Again, by design, participants correctly guessed the first colour on 80% of trials (there were 100 trials per unbalanced block). Of these, 64 were the more likely sequence, which would be guessed correctly at a probability of 80%. The other 16 trials would be the less likely sequence and should only be correctly guessed at a probability of 20% (i.e., 0.8*0.8*0.8 + 0.8*0.2*0.2 = 0.544). Actual performance on unbalanced blocks of trials was 56.1% (*SD* 5%).

We compared actual levels of performance on unbalanced blocks of trials against a prediction for a ‘no attempt to guess the second colour’ strategy (i.e., 64% correct) using a Bayes factor analysis. This delivered decisive evidence for the alternative hypotheses, that there would be a difference (*BF*_10_ > 1000). We also compared actual levels of performance on unbalanced blocks of trials against the prediction for a ‘guessing the second colour’ strategy (i.e., 54.4% correct). This revealed only anecdotal evidence for a difference in performance (*BF*_10_ = 1.226). Overall, these data provide strong evidence against participants simply choosing the more likely sequence on every trial of unbalanced blocks, although they might have guessed at the more likely outcome slightly more often than our task instructions encouraged (but we stress that evidence for this is only anecdotal).

## Discussion

We found that correctly guessing the first flash colour made it seem relatively protracted compared to when it was guessed incorrectly, but only if the second flash was guessed incorrectly (see Fig. 2A). We did not observe a shortening of perceived duration for repeated events (see Fig. 2B), or for statistically probable events (see Fig. 2C). We did observe a robust positive time-order error, with initial presentations of couplets seeming relatively protracted.

The purpose of this study was to combine multiple factors previously found to influence apparent duration into a single paradigm. We were interested in repetition (Pariyadath & Eagleman, 2007), statistical probability (Tse et al., 2004; Ulrich et al., 2006), guessing (Birngruber et al., 2018), and time-order errors (Rose & Summers, 1995). Of these, within our paradigm time-order error was the strongest influence on perceived duration. Of the other three factors, only declared predictions had an additional discernible impact on perceived duration.

In our data time-order errors were positive, with the first of a sequential pair of events appearing to have a longer duration. This might have been related to our protocol encouraging heightened attention to initial events, as the probability of guessing the entire sequence hinged on the outcome of the first event, potentially lessening the relevance of the second. Time order errors are more typically negative (e.g., Allan, 1977; Hellström, 1985); however, positive time-order errors have often been reported for very short test durations (< 1 s) similar to the test durations we have studied (Allan, 1977; Fraisse, 1948; Hellström, 1985; Needham, 1934; Vierordt, 1868). Similar reversed contingencies have also been found when judging sequentially lifted weights (Fechner, 1860; Woodrow, 1933) or when judging the intensities of sequential auditory experiences (Needham, 1935).

The finding that events that confirm declared predictions seem longer is consistent with the work of Birngruber et al. (2018). Moreover, this finding aligns with the theoretical framework proposed by Matthews (2015), wherein top-down attention can encourage enhanced responding to anticipated inputs. This hypothetical relationship is also supported by studies concerning the impact of attention on single-cell activity (Reynolds et al., 2000), and is reminiscent of confirmation bias (Bundesen, 1990; Nickerson, 1998; Rajsic et al., 2015). Bias-confirming events could trigger enhanced neural responding and an impression of a protracted event duration, as people monitor inputs for evidence that confirms their expectations.

There are other examples that tie attentional biases to duration dilations. Mattes and Ulrich (1998) showed that directing attention toward a future event, by cueing the modality in which it would occur, made the event seem longer. Drawing attention to the location of a brief event via exogenous (Enns et al., 1999) or endogenous cues (Seifried & Ulrich, 2010; Yeshurun & Marom, 2008), or by increasing certainty about when an event will occur (Grondin & Rammsayer, 2003; Wehrman et al., 2020), can also make events seem to last longer.

How might this relationship, between an attentional bias and enhanced neural processing leading to an exaggerated perceived duration, be operationalised? Contemporary evidence suggests that attention seems to be guided by a network of parietal and frontal areas, and by the superior colliculus (Bisley, 2011). In the visual cortex, neurons with receptive fields that encompass the spatial location of an attended stimulus synchronise their spiking at a gamma-band frequency (~35–90 Hz) (Fries et al., 2001; Tallon-Baudry et al., 2005; Womelsdorf & Fries, 2007), and desynchronise at lower frequency-spiking rates (Bauer et al., 2006; Fries et al., 2001; Yamagishi et al., 2005). The synchronisation strength of gamma frequency predicts behavioural response times to visual inputs (Gonzalez Andino et al., 2005; Womelsdorf et al., 2006), and perceptual accuracy (Taylor et al., 2005). Enhanced gamma synchronisation has also been shown to be triggered by feature-based attention in macaques (Bichot et al., 2005) and humans (Müller & Keil, 2004; Pavlova et al., 2006). This gamma increase could be important to the flow of sensory information into later cortical regions (Fries, 2009; van Kerkerle et al., 2014), resulting in more detailed processing of attended perceptual information.

In line with this, it has been shown that people are less sensitive to visual content that is expected due to a repetition-based prediction (Saurels et al., 2019), possibly because they attend less to them. If enhanced responding to declared predictions results from increased top-down directed attention (Matthews, 2015), we would expect people to be *more* visually sensitive these events. Consistent with this, it has been shown that people extract more information from events that are expected due to spatial or temporal cueing (Bausenhart et al., 2007; Carrasco & McElree, 2001). Future research could determine if this happens for predicted visual content from explicitly declared predictions.

Our data support the argument that the temporal oddball effect, as reported in many studies (e.g., Saurels et al., 2019; Tse et al., 2004), might be a product of several inter-related factors (Matthews, 2015). In standard temporal oddball protocols, participants are aware that a behavioural judgement will be required regarding repetition-violating events – but not for repeating events, which builds in an attentional bias that might act to exaggerate perceived oddball durations (Birngruber et al., 2015; Wehrman et al., 2020). The difference in apparent duration between oddballs and repeats in standard oddball presentation protocols could be enhanced when people are more certain they are about to see an oddball that requires judgement (Wehrman et al., 2020). This difference can be *diminished* by creating an attentional bias to a repeat event (Birngruber et al., 2015), demonstrating that the perceptual difference is largely (but not entirely) explained by a task demand that signals when attention will be required. This does not preclude an influence of repetition, which could give rise to reduced neural responding via low-level visual adaptation (Pariyadath & Eagleman, 2007; but also see Schindel et al., 2011).

The success of temporal oddball protocols, in contrast to the lack of a repetition effect in our data, might also suggest that multiple repetitions within a protocol are necessary to produce robust duration distortions contingent on repetition. Hence our data should not be taken as evidence that there is no repetition effect in standard temporal oddball protocols and experiments – just that there is no repetition effect in our data. The steps we took to experimentally tease apart the unique contributions of event probability, repetition, and declared predictions might have fundamentally changed the balance of factors that can contribute to subjective distortions of time. With that said, in our protocol the strongest influences on subjective duration were a positive time-order error (Rose & Summers, 1995), and the outcomes of declared predictions (Birngruber et al., 2018).

One might reasonably ask why we did not observe a difference in perceived duration for correct and incorrectly guessed second events. We speculate that this might have been due to opposing influences on second events in our task. One possibility is that participants simply paid more attention to the first flash (although the guessing behaviour data show that participants were motivated to guess the second flash colour correctly). Another possibility is that probable second events might have seemed shorter, but this effect could have been countered by an expansion of perceived time when declared predictions for second events were confirmed. As participants preferentially guessed the more probable event class, this was a common trial outcome. With this possibility acknowledged, we should also note that finding a temporal distortion for improbable events would contradict the findings of both Cai et al. (2015) and Matthews (2015).

An avenue for future research would be to examine multiple factors that encourage a statistical expectation within the same experiment (e.g., complex patterns and overlearned rules, such as the order of the alphabet). Such studies could also examine sensitivity to the content of events that are subject to differential temporal distortion. Moreover, the neural consequences of different sources of expectation could be compared using a modified version of our experimental protocol. Are early visual responses modified for prediction confirming inputs, consistent with top-down enhancement of processing (Matthews, 2015; Reynolds et al., 2000)?

In summary, this study has examined the effects of multiple factors that can contribute to the predictability of an event, and to human time perception: repetition (Pariyadath & Eagleman, 2007), statistical likelihood (Tse et al., 2004; Ulrich et al., 2006), guessing (Birngruber et al., 2018), and time-order errors (Hellström, 1985). We have observed that: (1) the first event of a sequential pair seems relatively protracted (a positive time-order error), (2) events that confirm a declared prediction tend to appear longer, but only if the next event does not also confirm a prediction, (3) neither stimulus repetition (two events), nor the likelihood of an event being presented, were sufficient to modulate perceived duration in our experiment. Overall, our data suggest that there is no simple mapping between event predictability and perceived duration. Rather, whether the predictability of an event will encourage a dilation or contraction of perceived time appears to depend on the origin of the expectation.
